# A phenome-wide association study (PheWAS) to identify the health impacts of 4-cresol sulfate in the Nagahama Study

**DOI:** 10.1038/s41598-023-40697-2

**Published:** 2023-08-25

**Authors:** Huiting Ou, Shuji Kawaguchi, Kazuhiro Sonomura, Takahisa Kawaguchi, Seri Kitada, Satoshi Yoshiji, François Brial, Dominique Gauguier, Jianguo Xia, Fumihiko Matsuda

**Affiliations:** 1 Center for Genomic Medicine, Graduate School of Medicine, Kyoto University, Kyoto, 606-8507 Japan; 2 Department of Human Genetics, McGill University, Montreal, QC H3A 0C7 Canada; 3 Life Science Research Center, Technology Research Laboratory, Shimadzu Corporation, Kyoto, 604-8511 Japan; 4 University Paris Cité, INSERM UMR1124, 45 rue des Saints Peres, 75006 Paris, France; 5 Institute of Parasitology, McGill University, Montreal, QC H9X 3V9 Canada

**Keywords:** Biomarkers, Diseases, Risk factors

## Abstract

Gut-microbiota derived metabolites are important regulators of host biology and metabolism. To understand the impacts of the microbial metabolite 4-cresol sulfate (4-CS) on four chronic diseases [type 2 diabetes mellitus, metabolic syndrome (MetS), non-alcoholic fatty liver disease, and chronic kidney disease (CKD)], we conducted association analyses of plasma 4-CS quantified by liquid chromatography coupled to mass spectrometry (LC–MS) in 3641 participants of the Nagahama study. Our results validated the elevation of 4-CS in CKD and identified a reducing trend in MetS. To delineate the holistic effects of 4-CS, we performed a phenome-wide association analysis (PheWAS) with 937 intermediate biological and behavioral traits. We detected associations between 4-CS and 39 phenotypes related to blood pressure regulation, hepatic and renal functions, hematology, sleep quality, intraocular pressure, ion regulation, ketone and fatty acid metabolisms, disease history and dietary habits. Among them, 19 PheWAS significant traits, including fatty acids and 14 blood pressure indices, were correlated with MetS, suggesting that 4-CS is a potential biomarker for MetS. Consistent associations of this gut microbial-derived metabolite on multiple endophenotypes underlying distinct etiopathogenesis support its role in the overall host health, with prospects of probiotic-based therapeutic solutions in chronic diseases.

## Introduction

The gut microbiota, composed of trillions of microorganisms^1,2^, participates in the host's biological processes such as nutrient metabolism, intestinal mucosal protection, neurological signalling, vitamin synthesis, and immunomodulation^3^. Recent research indicates that alternation in the microbial-derived metabolites plays a role in regulating host biology^4^ and susceptibility to various diseases^5^. For instance, elevated short-chain fatty acids (mainly butyrate, acetate, and propionate) reduce blood pressure in hypertensive patients^6^. On the other hand, trimethylamine-N-oxide increases the risk of cardiovascular risk^7^ and branched-chain amino acids (leucine, isoleucine, valine) are increased in diabetic and obese patients^8,9^.

Investigations into the pathophysiological roles of the gut-microbial metabolite 4-cresol have led to controversial conclusions. For decades, excessive accumulation of 4-cresol or its downstream metabolites has been consistently associated with chronic kidney disease (CKD) and deemed a uremic toxin^10,11^. Urinary levels of 4-cresol and its conjugated derivative 4-cresol sulfate (4-CS) are elevated in children with autism spectrum disorder (ASD)^12^. Nonetheless, studies indicated that increased levels of 4-CS reduce allergic airway responses relevant to asthma through decreased production of CCL20 in the airway epithelium^13^ and relieve inflammation in primary biliary cholangitis by mediating Kupffer cells^14^. Moreover, low blood levels of 4-cresol may benefit human health^15^. The metabolite was found to be negatively associated with type 2 diabetes mellitus (T2DM) in a Lebanese cohort, and chronic administration of low-dose of 4-cresol in preclinical models of T2DM resulted in reduced obesity and liver fat, improved glycemic control and enhanced insulin secretion and β-cell proliferation^15^.

Therefore, we hypothesized that 4-CS, the major conjugated metabolite of 4-cresol^16^ in humans, is linked to chronic diseases, including T2DM, metabolic syndrome (MetS), CKD, and non-alcoholic fatty liver disease (NAFLD). The ability of 4-cresol to reduce liver fat and to improve glycemic control^15^ prompted us to consider MetS, in which glucose intolerance and dyslipidemia are two major components^17^. In addition, NAFLD is a risk factor for CKD^18^, and insulin resistance is a hallmark in the pathogenesis of NAFLD, T2DM and MetS^19^. We first examined the relationship between 4-CS and these four disease endpoints in 3641 generally healthy Japanese individuals from the Nagahama study population^20^. Next, we took advantage of the extensive characterization of subjects of this cohort with multiple endophenotypes to uncover the roles of 4-CS in overall human health through the application of the phenome-wide association analysis (PheWAS)^21^ concept, by performing regression analyses between 4-CS and 937 human phenotypes. Significant associations related to blood pressure regulation, liver and kidney functions, sleep quality, intraocular pressure, ion regulation, fatty acid, and ketone metabolism were identified. Further analyses indicated that 4-CS could be connected to CKD via ion regulation and lipid regulation, whereas it may affect blood pressure, hematological, and liver regulations in MetS. Our results demonstrate the consistent impacts of 4-CS on multiple biological functions in healthy individuals and its association with indices of improved cardiometabolic health in humans.

## Results

### Among four chronic diseases, plasma 4-cresol sulfate is associated with CKD

Logistic regressions were performed to investigate the relationships between 4-CS and four chronic diseases, including T2DM, MetS, CKD, NAFLD. Population statistics for each disease are shown in Supplementary Table S1 online. The results indicate that plasma 4-CS is associated with CKD (OR = 1.26 [95% CI: 1.16, 1.37], *p* = 5.46 × 10^−8^). No evidence of statistically significant association was found between 4-CS and NAFLD (OR = 1.17 [95% CI: 0.87, 1.57], *p* = 0.31), nor MetS (OR = 0.99 [95% CI: 0.89, 1.10], *p* = 0.84), nor T2DM (OR = 1.02 [95% CI: 0.90, 1.15], *p* = 0.80) (Supplementary Fig. S1 online). In the Lebanese study, where plasma 4-cresol was negatively associated with T2DM, a notable characteristic of this population was the extreme symptoms of cardiometabolic diseases, and the participants were overweight or obese^15^. To mimic such conditions, only obese T2DM participants (n = 65) were selected to compare with control individuals. Again, the logistic regression indicates the absence of significant association between 4-CS and T2DM (OR = 0.90 [95% CI: 0.71, 1.14], *p* = 0.38).

Since blood 4-CS levels could be influenced by environmental factors such as diet and medication intake, we repeated statistical analyses following adjustment for additional covariates in order to re-examine 4-CS’ effects on T2DM. Results showed no evidence of statistical significance between 4-CS and T2DM (OR = 0.99 [95% CI: 0.87, 1.13], *p* = 0.90). Furthermore, we investigated the impacts of 4-CS in non-T2DM individuals (n = 3279) on T2DM-related indices, including blood levels of glucose, insulin, and hemoglobin A1c (HbA1c), as well as the homeostasis model assessment of insulin resistance (HOMA-IR). Again, we did not identify evidence of statistical significance between 4-CS on blood glucose (β = 0.01 [95% CI: − 0.02, 0.04], *p* = 0.06), blood insulin (β = − 0.01 [95% CI: − 0.05, 0.02], *p* = 0.44), blood HbA1c (β = − 0.03 [95% CI: − 0.05, 1.04 × 10^−3^], *p* = 0.06) and HOMA-IR (β = − 0.02 [95% CI: − 0.06, 0.01], *p* = 0.21).

### 4-Cresol sulfate shows evidence of significant associations with multiple phenotype categories

**Figure 1:**
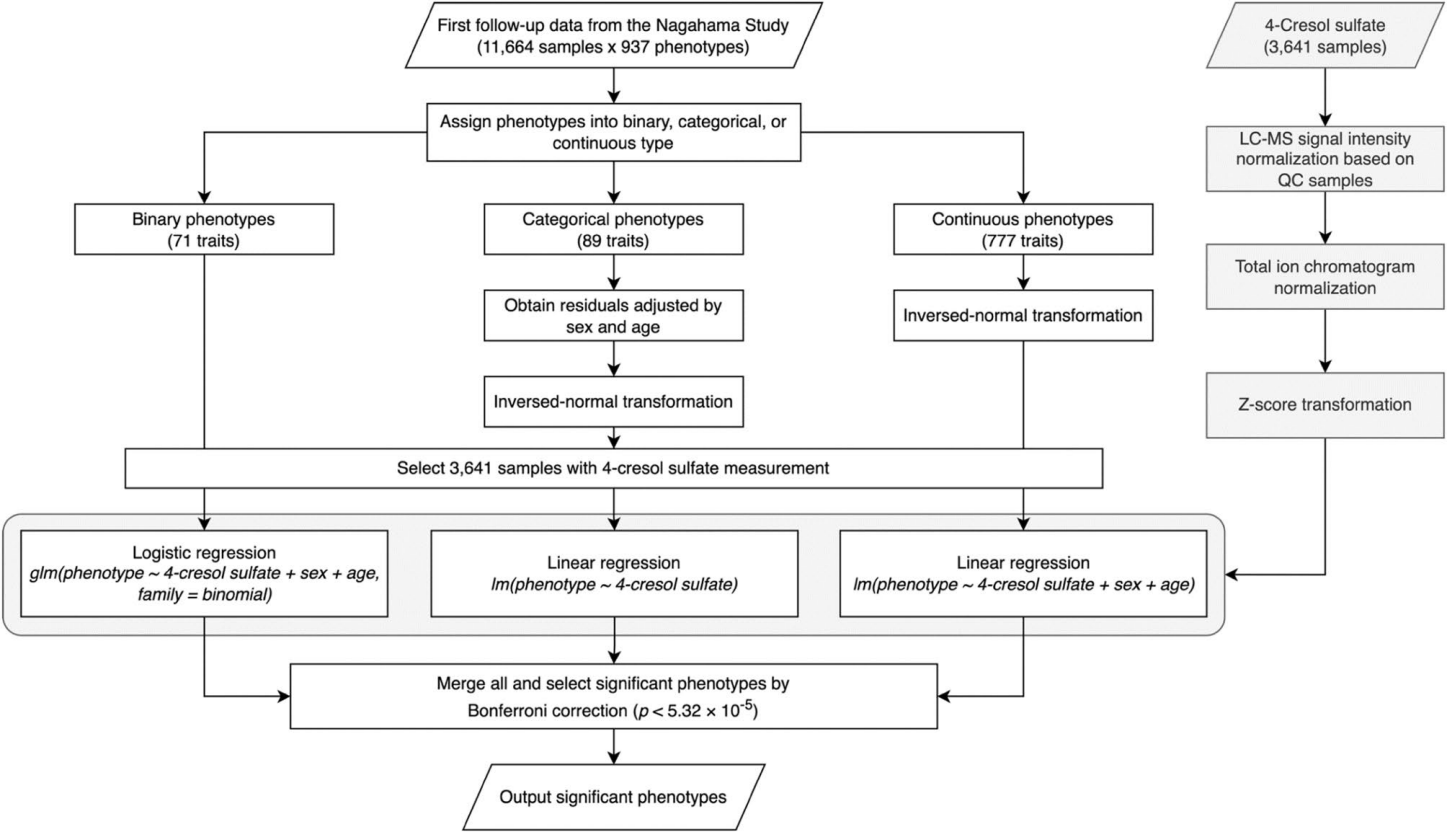
Details of the PheWAS analysis pipeline. This pipeline was constructed in the R language with Nextflow.

**Figure 2:**
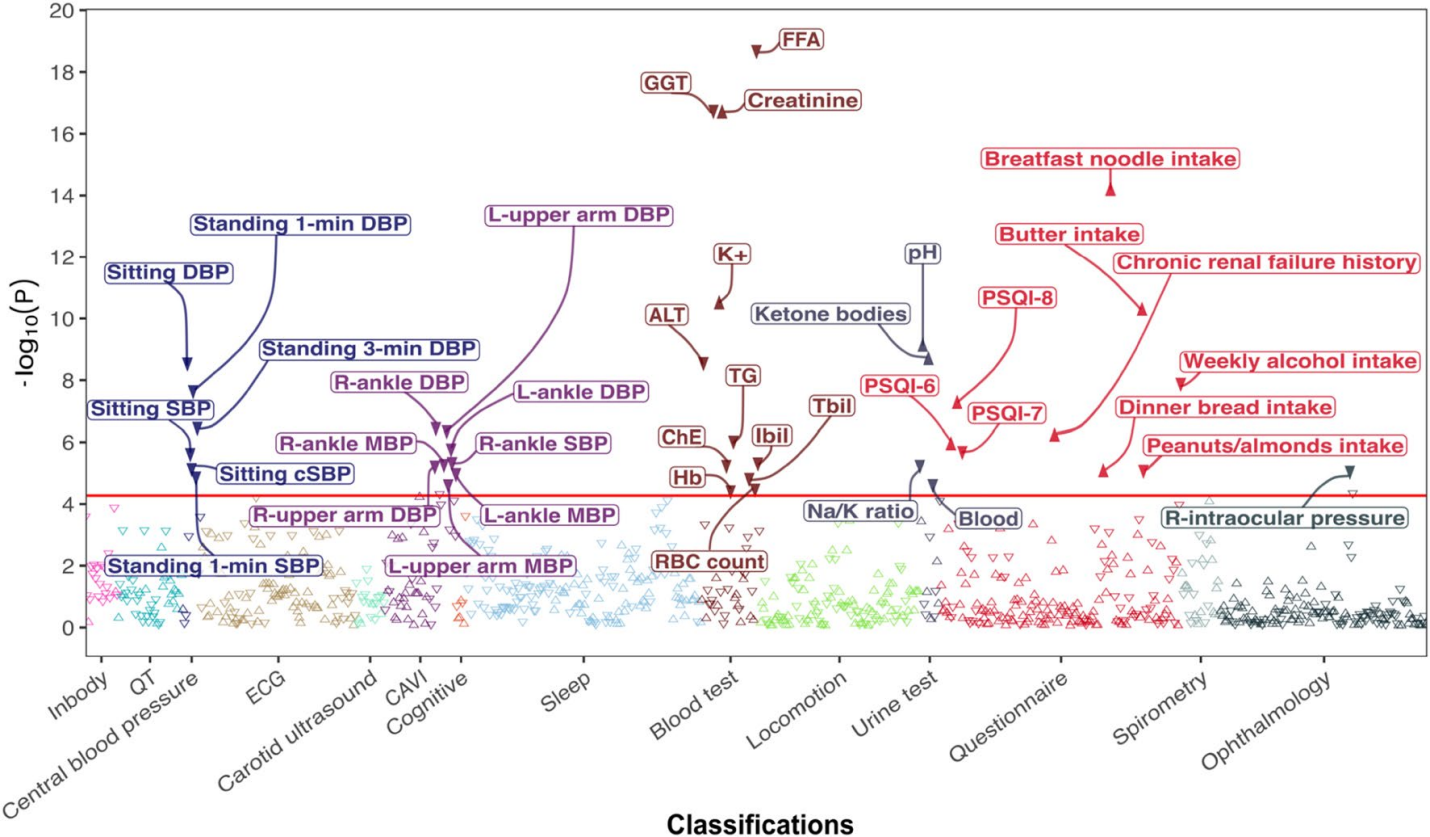
Manhattan plot showing the 39 intermediate phenotypes associated with plasma 4-cresol sulfate through PheWAS in the Nagahama study population. A total of 937 intermediate phenotypes collected in 3641 generally healthy individuals of the Nagahama Study were used to test for significant association (*p* < 5.32 × 10^−5^) with 4-cresol sulfate. *ALT* alanine aminotransferase, *CAVI* cardio‐ankle vascular index, within which L denotes left, R denotes right, *ChE* cholinesterase, *ECG* electrocardiogram, *GGT* gamma-glutamyl transferase, *FFA* free fatty acids, *Hb* hemoglobin, *Ibil* indirect bilirubin, *K*+ potassium level, *Tbil* total bilirubin, *QT* QT-interval, *RBC count* red blood cell count, *R-intraocular pressure* right intraocular pressure corrected by corneal pressure, *TG* triglycerides.

To examine the holistic biological effects of 4-CS, we conducted a PheWAS (Fig. 1) with 937 intermediate phenotypes distributed in 14 categories (Table 1). Regression analysis results (Supplementary Table S2 online) indicated evidence of statistically significant associations between plasma 4-CS and 39 endophenotypes (Fig. 2, Table 2) from five categories: 8 from the cardio-ankle vascular index (CAVI), 6 from central blood pressure, 11 from the blood test, 4 from the urine test, 1 from ophthalmology, and 9 from the questionnaires. In the CAVI category, the phenotypes significantly associated with 4-CS included 14 blood pressure indices: right upper arm diastolic blood pressure (DBP), left upper arm DBP, left upper arm mean blood pressure (MBP), right ankle systolic blood pressure (SBP), right ankle DBP, right ankle MBP, left ankle DBP and left ankle MBP. The significant central blood pressure traits include sitting central SBP (cSBP), sitting SBP, sitting DBP, standing 1-min SBP, standing 1-min DBP and standing 3-min DBP. All 14 blood pressure traits were consistently negatively associated with 4-CS.

**Table 1:** Detailed categorization of the 937 intermediate phenotypes tested for evidence of association with plasma 4-cresol sulfate in the Nagahama Cohort.

Classification	Description	Number of traits
Inbody	Muscle mass, body fat, mineral, etc	23
Blood test	Ion levels, hemoglobin, insulin, cholesterol, fatty acids, protein, glucose, blood cell counts, etc	41
Urine test	Protein, blood cell sediment, ions levels, glucose, etc.	15
QT	QT-heart ECG derived measurements	45
Central blood pressure	Blood pressure measurements of standing and sitting	13
ECG	ECG waveform	108
Carotid ultrasound	Blood flow through the carotid arteries	20
CAVI	Blood pressure indices	50
Cognition	Cognitive impairment and the Nagahama grading	7
Locomotion	Walking data and knee movement etc.	111
Ophthalmology	Eye pressure, eye length, eye thickness etc.	145
Questionnaire	Knee pain, sleep quality (PSQI), disease history, pets, skin conditions, living conditions, medications, education, smoking details, income, alcohol intake etc.	168
Spirometry	Lung and breathe-related measurements	27
Sleep data	Sleep time, wake-up time, sleep duration etc	164

*CAVI* cardio-ankle vascular index, *ECG* electrocardiogram, *QT* QT-interval.

**Table 2:** Summary statistics of association results, including 4-cresol sulfate’s association significant traits, four chronic diseases’ associations with the thirty-nine 4-cresol sulfate related phenotypes. For clarity, the blood pressure intermediate phenotype with the lowest p-value for 4-cresol sulfate is displayed. Details for the remaining 13 blood pressure traits are shown in Supplementary Table S4 online. Creatinine was excluded for chronic kidney disease (CKD). Full details, including all results for the four chronic diseases are shown in Supplementary Table S4 online. *MetS* metabolic syndrome, *CI* confidence interval. *OR* odds ratio.

Biological functions	Phenotype names	4-Cresol sulfate		CKD		MetS	
		Beta (95% CI)	p-value	OR (95% CI)	p-value	OR (95% CI)	p-value
Blood pressure regulation	Sitting DBP	− 0.09 (− 0.12, − 0.06)	3.51 × 10^−9^	1.00 (0.99, 1.01)	0.54	1.03 (1.02, 1.04)	1.88 × 10^−8^
Dietary habit	Breakfast noodle intake	0.12 (0.09, 0.14)	6.92 × 10^−15^	1.03 (0.85, 1.25)	0.77	1.16 (0.95, 1.41)	0.14
	Dinner bread intake	0.06 (0.04, 0.09)	9.07 × 10^−6^	0.94 (0.77, 1.15)	0.56	0.94 (0.75, 1.18)	0.59
	Butter intake	0.10 (0.07, 0.12)	5.52 × 10^−11^	0.99 (0.89, 1.10)	0.86	1.00 (0.88, 1.13)	1
	Peanuts/almonds intake	− 0.07 (− 0.10, − 0.04)	1.02 × 10^−5^	1.04 (0.95, 1.13)	0.39	0.94 (0.84, 1.04)	0.23
	Weekly alcohol intake	− 0.06 (− 0.09, − 0.04)	1.60 × 10^−8^	0.99 (0.97, 1.00)	0.14	1.00 (0.98, 1.02)	1
Disease history	Chronic renal failure history	1.92 (1.49, 2.48)	6.44 × 10^−7^	10.79 (2.74, 42.46)	6.68 × 10^−4^	3.46 (0.75, 15.98)	0.11
Fatty acid metabolism	Triglycerides	− 0.07 (− 0.10, − 0.04)	1.17 × 10^−6^	1.003 (1.002, 1.005)	1.14 × 10^−4^	1.01 (1.01, 1.01)	5.64 × 10^−26^
	Free fatty acids	− 0.14 (− 0.17, − 0.11)	2.56 × 10^−19^	0.60 (0.34, 1.03)	0.06	2.18 (1.19, 3.97)	0.01
Hematology	Red blood cell count	− 0.06 (− 0.08, − 0.03)	4.13 × 10^−5^	1.00 (0.996, 1.00)	0.37	1.01 (1.00, 1.01)	2.61 × 10^−4^
	Hemoglobin	− 0.05 (− 0.07, − 0.03)	4.71 × 10^−5^	0.90 (0.82, 0.99)	0.03	1.24 (1.11, 1.38)	1.37 × 10^−4^
Intraocular Pressure	Right intraocular pressure corrected by corneal pressure	− 0.07 (− 0.10, − 0.04)	1.09 × 10^−5^	1.00 (0.96, 1.03)	0.84	1.06 (1.02, 1.10)	4.74 × 10^−3^
Ion regulation	Urinary pH	0.09 (0.06, 0.12)	8.06 × 10^−10^	0.81 (0.72, 0.92)	1.37 × 10^−3^	1.01 (0.88, 1.16)	0.89
	Urinary Na^+^/K^+^ ratio	− 0.07 (− 0.10, − 0.04)	7.06 × 10^−6^	0.87 (0.81, 0.94)	2.41 × 10^−4^	1.08 (1.01, 1.15)	0.02
	Potassium level	0.10 (0.07, 0.13)	3.41 × 10^−11^	3.18 (2.33, 4.32)	2.20 × 10^−13^	0.62 (0.43, 0.89)	0.01
Ketone metabolism	Urinary ketone bodies	0.09 (0.06, 0.12)	2.16 × 10^−9^	NA	NA	NA	NA
Liver function	Total bilirubin	− 0.06 (− 0.09, − 0.03)	1.87 × 10^−5^	0.55 (0.35, 0.84)	0.01	0.73 (0.47, 1.14)	0.16
	Indirect bilirubin	− 0.07 (− 0.09, − 0.04)	5.90 × 10^−6^	0.45 (0.25, 0.79)	0.01	0.60 (0.33, 1.09)	0.09
	Alanine aminotransferease	− 0.08 (− 0.11, − 0.06)	3.41 × 10^−9^	1.00 (0.99, 1.01)	0.4	1.02 (1.01, 1.03)	6.41 × 10^−10^
	Gamma-glutamyl transferase	− 0.12 (− 0.14, − 0.09)	2.23 × 10^−17^	1.00 (0.99, 1.00)	0.32	1.00 (1.00, 1.01)	3.79 × 10^−4^
	Cholinesterase	− 0.07 (− 0.10, − 0.04)	7.10 × 10^−6^	1.00 (0.998, 1.00)	0.24	1.01 (1.00, 1.01)	4.09 × 10^−13^
Renal function	Urinary blood	− 0.07 (− 0.10, − 0.04)	2.95 × 10^−5^	1.13 (0.96, 1.32)	0.13	0.93 (0.76, 1.14)	0.48
	Creatinine	0.10 (0.07, 0.12)	2.16 × 10^−17^	NA	NA	4.48 (1.97, 10.20)	3.58 × 10^−4^
Sleep quality	PSQI-6 Sleep quality	0.07 (0.04, 0.10)	1.24 × 10^−6^	0.83 (0.69, 1.00)	0.05	1.37 (1.12, 1.67)	2.15 × 10^−3^
	PSQI-7 Use of sleeping pills	0.08 (0.05, 0.11)	5.72 × 10^−8^	0.85 (0.69, 1.05)	0.13	1.17 (0.97, 1.41)	0.09
	PSQI-8 Daily sleepiness	− 0.08 (− 0.11, − 0.04)	2.52 × 10^−6^	1.05 (0.94, 1.17)	0.36	1.00 (0.87, 1.14)	0.95

The 11 blood traits associated with 4-CS include total bilirubin, indirect bilirubin, free fatty acids, triglycerides, creatinine, alanine aminotransferase, gamma-glutamyl transferase, potassium level, hemoglobin, red blood cell count, and cholinesterase. The urinary traits significantly associated with 4-CS include Na^+^/K^+^ ratio, levels of ketone body, and pH (Fig. 2, Table 2). As for the ophthalmological traits, the intraocular pressure adjusted by corneal pressure was negatively associated with plasma 4-CS. Moreover, we identified significant associations between 4-CS and three sleeping phenotypes from the Pittsburgh Sleep Quality Index (PSQI), including PSQI-6 for sleep quality, PSQI-7 for sleeping medication intake, and PSQI-8 for daily sleepiness (Fig. 2). Elevated plasma 4-CS was correlated with worsening sleep quality (PSQI-6), increased sleeping pills intake (PSQI-7) and lower frequency of daytime sleepiness (PSQI-8) (Table 2). Five dietary habits were significantly associated with 4-CS, including three positive associations with butter consumption, breakfast noodle intake frequency and the frequency of dinner bread intake, and two negative associations with alcohol and peanuts or almonds consumption. Lastly, histories of renal failure were positively associated with 4-CS concentration (Table 2).

Results from PheWAS uncover multiple significant associations of 4-CS with a priori biologically unrelated variables (e.g., sleep quality, dietary habits, ion regulation). This broad-ranging pattern of associations suggests coordinately regulated biological mechanisms and pathways by 4-cresol, with possible consequences on the risk of several chronic disorders.

### MetS and CKD are significantly associated with multiple 4-CS-related traits

To further identify the endophenotypes related to the four chronic diseases, we conducted regression tests between the thirty-nine traits significantly associated with 4-CS and the four chronic disease endpoints by logistic regression. The statistics of these 39 traits in each disease are shown in Supplementary Table S3 online. After Bonferroni correction, T2DM and NAFLD showed no evidence of significant correlations with any phenotypes. Nonetheless, CKD was associated with three endophenotypes: positively associated with triglyceride (OR = 1.003 [95% CI: 1.002, 1.005], *p* = 1.14 × 10^−4^) and potassium levels (OR = 3.18 [95% CI: 2.33, 4.32], *p* = 2.20 × 10^−13^) and negatively associated with the urinary Na^+/^K^+^ ratio (OR = 0.87 [95% CI: 0.81, 0.94], *p* = 2.41 × 10^−4^). MetS was positively associated with 19 traits, including 14 blood pressure indices (right upper arm DBP, right ankle DBP, right ankle MBP, right ankle SBP, left upper arm DBP, left upper arm MBP, left ankle DBP, left ankle MBP, sitting DBP, sitting SBP, sitting cSBP, standing 1-min DBP, standing 1-min SBP, standing 3-min DBP), triglycerides, two hematological traits (hemoglobin and red blood cell count) and two liver indicators (alanine aminotransferase and cholinesterase) (Table 2, Supplementary Table S4 online).

## Discussion

This study elucidates the functional relationships between the gut microbial metabolite 4-CS and multiple phenotypes in healthy individuals. We report broad-ranging associations between 4-CS and liver function, sleep quality, intraocular pressure, ion regulation, dietary habits, blood pressure regulation and ketone and fatty acids metabolisms, the latter three suggesting its impact on cardiometabolic risk. Further detailed analyses on four disease endpoints identified a positive association between 4-CS and CKD and a reducing trend with MetS risk.

Our findings of elevated 4-CS in CKD cases confirm its well-known characteristic as a uremic toxin^10,16,22^. The biological mechanisms and pathways contributing to high 4-CS and clinical consequences are reviewed in detail^22^. Abnormal potassium metabolism and renal Na^+^/K^+^ transport increase the risk of developing CKD^23^. The concordance of associations of 4-CS with CKD and both serum potassium and urine Na^+^/K^+^ ratio in our study highlight the importance of electrolyte homeostasis in CKD. This is consistent with a previous study which indicated that 4-cresol impacted Na^+^/K^+^-ATPase’s activity in the rat brain^24^. Therefore, ion regulation might be the biological mechanism underlying the contribution of 4-CS to CKD risk.

Despite the absence of statistically significant association with NAFLD, 4-CS was associated with decreased liver indices, including alanine aminotransferase, gamma-glutamyl transferase, cholinesterase and total bilirubin, suggesting improved hepatic function. Thus far, few studies have reported a role of 4-CS in liver biology. In a mouse study of kaempferol’s beneficial effects in non-alcoholic steatohepatitis (NASH), a progressive type of NAFLD, serum 4-CS was higher in both kaempferol-treated and control mice as compared to the diseased mice^25^. Another study showed that 4-CS could reduce inflammation in primary biliary cholangitis mice, but caused liver damage in normal mice^14^. Further investigations are required to understand the role of 4-CS in liver function.

The lack of significant association of 4-CS with T2DM contrasts with the previous findings where serum 4-cresol was negatively associated with T2DM in the Lebanese population^15^. Pathophysiological characteristic dissimilarities in the two cohorts may account for this contradiction: the Lebanese case–control study dealt with subjects who had developed evidence of cardiometabolic disorders, whereas the Nagahama study population includes generally healthy Japanese individuals. Nonetheless, 4-CS may have greater impacts on the obese subtype of T2DM. Indeed, a case–control study reported decreased urine 4-cresol levels in T2DM patients^26^. Further classification of these T2DM patients into two subtypes indicated that the diabetic subtype with low 4-cresol levels showed evidence of higher BMI, body weight, waist circumference, and lower plasma glucose^26^, which is consistent with our speculation. In addition, results from statistical analyses after adjustment for environmental variables such as diet and medication failed to identify an impact of 4-CS on T2DM and T2DM-related traits in non-T2DM individuals.

Despite the insignificant association between 4-CS and MetS, the odds ratio indicated an inversed trend. MetS is a disorder characterized by a combination of four major risk factors, including glucose intolerance, obesity, dyslipidemia, and hypertension^27^. Currently, multiple diagnostic criteria co-exist depending on the geographical regions^28^. In our study, obesity was a mandatory criterion for MetS diagnosis according to the Japanese Society of Internal Medicine Guideline^29^. Therefore, our results may reflect a specific MetS type. Additional analyses of the 39 PheWAS traits significantly associated with MetS indicate the potential health benefits of 4-CS. First, its negative correlation with 14 blood pressure indices indicates a relationship between elevated plasma 4-CS and reduced blood pressure, which, to our knowledge, is a novel observation in the healthy population. Interestingly, infusion of angiotensin II designed to lower hypertension in mice bred in conventional conditions of maintenance resulted in increased plasma 4-CS as compared to germ-free mice^30^, indicating a similar trend. Nonetheless, the role of 4-CS in the cardiovascular system in healthy individuals remains elusive. Secondly, we found a negative association between plasma 4-CS and triglycerides, pointing to obesity reduction. This aligns with decreased liver triglycerides and adiposity in obese mice treated with 4-cresol^15^. Furthermore, a study showed that 4-CS inhibits lipogenesis in 3T3-L1 preadipocytes and in isolated human adipocytes^22^. Thirdly, two hematological traits (hemoglobin^31^ and red blood cell counts^32^) and two liver indicators (alanine aminotransferase^33^ and cholinesterase^34^), which are elevated in MetS, showed inversed relationships with 4-CS in our study. In summary, we hypothesize that 4-CS might be a potential biomarker of MetS prediction in healthy individuals.

The depth of phenotypic variations in PheWAS allowed us to identify relationships between plasma 4-CS and lifestyle choices, mainly sleep quality and dietary habits. Previous investigations of sleep quality with this metabolite have generated inconsistent results^35^. Urine 4-CS is decreased in situations of acute sleep deprivation^36^, whereas we observed associations between elevated plasma 4-CS and low sleep quality and increased usage of sleeping pills, suggesting that impaired elimination of 4-CS from plasma to urine may account for modified sleep quality. In addition, dialysis patients with excessive blood 4-CS reported lower sleep quality^37^. Finally, the dietary choices, including saturated fat present in butter or intake of noodles and bread, may also underlie the effect of food sources rich in 4-cresol^12^.

Our results imply a functional link between the alternation of the gut microbiota that enhances or reduces the production or availability of 4-cresol to the host for chronic diseases. The plausible ways dietary intake alters the level of 4-CS in the host are increasing the consumption of food sources enriched with this metabolite and/or adjusting the gut environment favourable for intestinal expansion of 4-cresol-producing bacteria, such as Bacteroidaceae and Clostridium clusters^38^. These data further support the importance of the metabolic function of the gut microbiome in the susceptibility of individuals to developing chronic diseases and the opportunities to design nutritional and probiotic-mediated solutions that stimulate 4-CS levels in treating this cluster of metabolic diseases.

Several limitations exist in this study. First, diagnostic accuracy of the three chronic diseases (T2DM, CKD and NAFLD) might be compromised. Of note, T2DM was determined by self-answered questionnaires, CKD diagnosis was based solely on creatinine level at a single time point, and NAFLD cases were defined only by the FIB-4 score. Nevertheless, board-ranging intermediate phenotypes systematically available for all subjects in the Nagahama population study have provided important clues for analysis of disease risks. Second, association analyses were performed with few covariates and cannot assess causality nor determine the beneficial range of 4-CS required to avoid any toxic effects. Therefore, future mechanistic analysis carefully accounting for appropriate covariates and Mendelian randomization studies with genetic information are required to test our hypotheses. Finally, replication in large population studies with various ethnic backgrounds should also be conducted before generalization.

## Conclusions

Results from our PheWAS illustrate a cost-effective approach to study the systemic biological function of 4-CS^21^. To our knowledge, this is the first PheWAS targeting a disease-predicting candidate metabolite, presenting a new analytical method for metabolite analysis. Our work provides supportive evidence of the beneficial role of 4-cresol metabolism on host health and continues challenging the impact of 4-CS, a long-regarded uremic toxin, in healthy individuals. We propose that non-toxic levels of this metabolite can positively impact human health. Extension of the phenotype screening to include unexplored organ systems enables multiple hypotheses generation, thus providing a high-level overview of the possible roles of this metabolite in humans. Overall, our study supports the notion that microbial-derived metabolites can affect not only metabolism but also the overall health of the host and promote the development of novel therapeutic solutions for chronic diseases.

## Materials and methods

### The Nagahama Study

Data were obtained from the first follow-up of the Nagahama Cohort for the Comprehensive Human Bioscience, a community-based prospective cohort study conducted in Nagahama City in Shiga prefecture, Japan. A total of 11644 middle-aged to elderly residents participated in the first follow-up from 2012 to 2016. Initially, there were 3645 individuals whose metabolome data were measured. Four individuals without available phenotype information were excluded. Therefore, 3641 individuals were included in this analysis. The details of this cohort have been reported elsewhere^20^. This study was conducted in accordance with the principles of the Declaration of Helsinki and was approved by the ethics committee of Kyoto University Graduate School of Medicine and by the Nagahama Municipal Review Board (no. 278). Written informed consent was obtained from all participants.

### Plasma preparation and 4-cresol sulfate measurement

Liquid chromatography coupled to mass spectrometry (LC–MS) was used to quantify 4-cresol sulfate (4-CS) in plasma samples. Peripheral blood samples were collected in 7 ml EDTA vacutainers (Venoject II, VP-NA070K, Terumo, Tokyo, Japan), immediately stored in a CubeCooler (Forte Grow Medical Co. Ltd., Tochigi, Japan) and kept at 4 °C until the centrifugation at 4 °C at 3000 rpm for 15 min. All the harvested plasma samples were then stored at − 80 °C until analysis. Fifty μl of plasma was mixed with 250 μl of methanol and the obtained mixture was shaken at 1200 rpm for 10 min at 37 °C (Maximizer MBR-022UP, Taitec). After centrifugation at 16,000×*g* for 30 min at 25 °C, 150 μl of supernatant was mixed with 90 μl of 1% acetic acid in water and 120 μl of chloroform, followed by a vortex mixing for 15 s. After centrifugation at 2000×*g* for 10 min at 25 °C, 150 μl of the upper layer was dried and solubilized in 50 μl of 0.1% formic acid in water, and then subjected to LC–MS analysis. LC separation was conducted on a Shim-pack GIST C18-AQ column (3 μm, 150 mm × 2.1 mm id, Shimadzu GLC) with a Nexera UHPLC system (Shimadzu). The mobile phase consisted of 0.1% formic acid in water (A) and 0.1% formic acid in acetonitrile (B). The gradient program was as follows: 0–3 min, 0% B; 3–15 min, linear gradient to 60% B; 15–17.5 min, 95% B; 17.5–20.0 min, linear gradient to 0% B; hold for 4 min; flow rate, 0.2 ml/min. The column oven temperature was maintained at 40 °C. The LC system was coupled with a triple-quadruple mass spectrometer LCMS-8060 (Shimadzu). LCMS-8060 was operated with the electrospray ionization and multiple reaction monitoring mode. Metabolites were measured for 3645 individuals with pooled quality control (QC) samples. We generated two pools and inserted two QC samples between intervals of five to nine Nagahama samples (a total of 743 QC samples). Peak intensities for each ion transition were extracted by LabSolutions LCMS software (Version 5.53 SP3, Shimadzu, Japan). The ion transition (precursor–product ion) for 4-CS was m/z 187.0–107.1. The extracted peak intensities were normalized based on QCs to address signal drifts and batch effects (see Supplementary Information for details), scaled by total ion chromatogram, and then transformed by the z-score.

### Ascertainment of chronic kidney disease (CKD)

The status of CKD was determined by the estimated glomerular filtration rate (eGFR) level at a single time point. The eGFR was calculated by eGFR [mL/min/1.73 m^2^] = 194 × (serum creatinine [mg/dL])^−1.094^ × age [years]^−0.287^ × 0.739 (if female). CKD was defined as eGFR < 60 ml/min/1.73 m^2^, resulting in 425 cases and 3216 controls.

### Ascertainment of type 2 diabetes mellitus (T2DM)

The status of T2DM was determined by web-based questionnaires, hemoglobin A1c (HbA1c) level, fasting hours, and glucose concentration. In the web-based questionnaire, participants who indicated they were previously diagnosed with type 1 diabetes (n = 14) and gestational diabetes (n= 10) were excluded. In addition, participants were required to answer the question “Have you had type 2 diabetes mellitus?”, the responses were chosen from one of the following answers: “No disease” (n = 3352), “Diagnosed, under treatment” (n = 200), “Completed treatment, no symptoms now” (n = 13), “Diagnosed once, but not consulting doctors” (n = 75). To eliminate self-reporting bias, only participants who chose “No disease” (n = 3352) or “Diagnosed, under treatment” (n = 200) were included. Furthermore, “No disease” individuals with glucose ≥ 126 mg/dl after ≥ 8 h after a meal (n = 1) or HbA1c ≥ 6.5% (n = 48) or both (n = 8) or low kidney function defined as eGFR < 30 (n = 6) were excluded. After all filtering criteria, the remaining participants were categorized as controls for “No disease” (n = 3279) and cases for “Diagnosed, under treatment” (n = 191). The four T2DM-related traits are blood levels of glucose, insulin, HbA1c, and the homeostasis model assessment of insulin resistance (HOMA-IR). HOMA-IR was calculated by HOMA-IR = fasting insulin [μIU/ml] × fasting glucose [mg/dl]/405 for individuals whose time after the last meal ≥ 8 h based on the questionnaire. For the analysis of the four T2DM-related traits, the sample size is 3279, except for HOMA-IR (n = 2509).

### Ascertainment of metabolic syndrome (MetS)

The diagnosis of MetS was based on the criteria from the Japanese Society of Internal Medicine in 2005^29^. Specifically, a diseased individual must have an abdominal circumference ≥ 90 cm for females or ≥ 85 cm for males. Besides, one must meet two out of three following clinical conditions: (1) triglyceride level ≥ 150 mg/dl or high-density lipoprotein (HDL) cholesterol < 40 mg/dl or on anti-hyperlipidemic medications. (2) systolic blood pressure (SBP) ≥ 130 mmHg and/or diastolic blood pressure (DBP) ≥ 85 mmHg or on anti-hypertensive medications. (3) fasting plasma glucose ≥ 110 mg/dl (10 h or more fasting time) or on diabetes medications. Applying these diagnostic criteria to the 3460 individuals previously selected for T2DM, 326 individuals were assigned as cases and 3144 as controls.

### Ascertainment of non-alcoholic fatty liver disease (NAFLD)

Individuals who have drinking habits were eliminated (n = 1927). The Fibrosis-4 (FIB-4) score was calculated by FIB-4 = (age [years] × aspartate transaminase (AST) [IU/L]/(platelet count [10^9^/L] × √ alanine aminotransferase (ALT) [IU/L]). To be classified as a NAFLD case, one must have a FIB-4 > 2.67. The NAFLD control group was defined as FIB-4 < 1.30. Individuals whose Fib-4 score was 1.30 and 2.67 were eliminated to avoid ambiguity. Finally, the number of cases and controls were 116 and 667, respectively.

### Phenotype description

A total of 937 phenotypes were integrated, representing the following health aspects: self-reported disease history and lifestyle-related questionnaires, blood test results on hematology and immunological indices, urinary traits, physiological reports on the cardio-ankle vascular index (CAVI), central blood pressure, spirometry, electrocardiogram, dental examinations, ophthalmological phenotypes, sleep information, brain MRI data, cognitive tests, and athletic activity (Table 1).

### Phenome-wide association analysis (PheWAS)

The detailed PheWAS pipeline is shown in Fig. 1. All 937 phenotypes were classified into three types: 71 binary, 89 ordinal, and 777 continuous phenotypes. Logistic regression was conducted between binary traits and 4-CS adjusted by sex and age. For the ordinal phenotypes, they were first adjusted by sex and age to obtain residuals. The residuals were normalized by inversed-normal transformation. Linear regression was conducted between the transformed data and 4-CS concentration. Continuous phenotypes were first transformed by inversed-normal transformation. Linear regression was performed between each continuous trait and 4-CS with sex and age as covariates. Among 937 phenotypes, the significant ones were selected by p-values less than the Bonferroni correction threshold *p* < 5.34 × 10^−5^.

### Association analysis with four chronic diseases

To investigate the association between 4-CS and four metabolic diseases, logistic regression was conducted between each disorder and 4-CS with sex and age as covariates. For T2DM, we adjusted for environmental effects on 4-CS by adding additional covariates as follows: among all medications and dietary traits, five 4-CS-associated traits (butter consumption, breakfast noodle intake frequency and the frequency of dinner bread intake, alcohol intake, and peanuts or almonds consumption) were identified. These five traits, plus eGFR level and the time after participants’ last meal when the blood sample was drawn modelled by spline function using 4-CS as the response, were included in the logistic regression model besides sex and age. To investigate 4-CS’ effects on the four T2DM-related traits (blood levels of glucose, insulin, HbA1c, and HOMA-IR) in the control populations, data for non-T2DM individuals were analyzed. These four T2DM-related traits were inversed-normal transformed. Linear regressions were conducted between normalized T2DM-related traits and 4-CS with covariates listed in the logistic regression model for adjusting environmental effects.

To identify the relationships between 4-CS-associated phenotypes and four metabolic diseases, logistic regressions were performed between the 4 chronic diseases and thirty-nine 4-CS-associated phenotypes adjusted for sex and age. Bonferroni correction (*p* < 3.22 × 10^−4^) was applied to obtain statistical significance. Statistical analysis was performed under R environment version 4.0.3.

### Supplementary Information

Supplementary Figure S1.

Supplementary Table S1.

Supplementary Table S2.

Supplementary Table S3.

Supplementary Table S4.

Supplementary Information

## Data Availability

All datasets and scripts used in this analysis are available from the corresponding author upon reasonable request.
