# Supplementary material for: A phenome-wide association study (PheWAS) to identify the health impacts of 4-cresol sulfate in the Nagahama Study
Source: Sci Rep. 2023 Aug 25;13:13926. doi: 10.1038/s41598-023-40697-2 (PMC10457396; doi:10.1038/s41598-023-40697-2)

Supplementary Figure S1. 4-cresol sulfate levels in cases and controls for four chronic diseases, a. Non-alcoholic fatty liver disease (NAFLD), b. Chronic kidney disease (CKD), c. Metabolic syndrome (MetS), d. Type 2 diabetes mellitus (T2DM), e. Obese T2DM. p-values were generated by logistic regression.

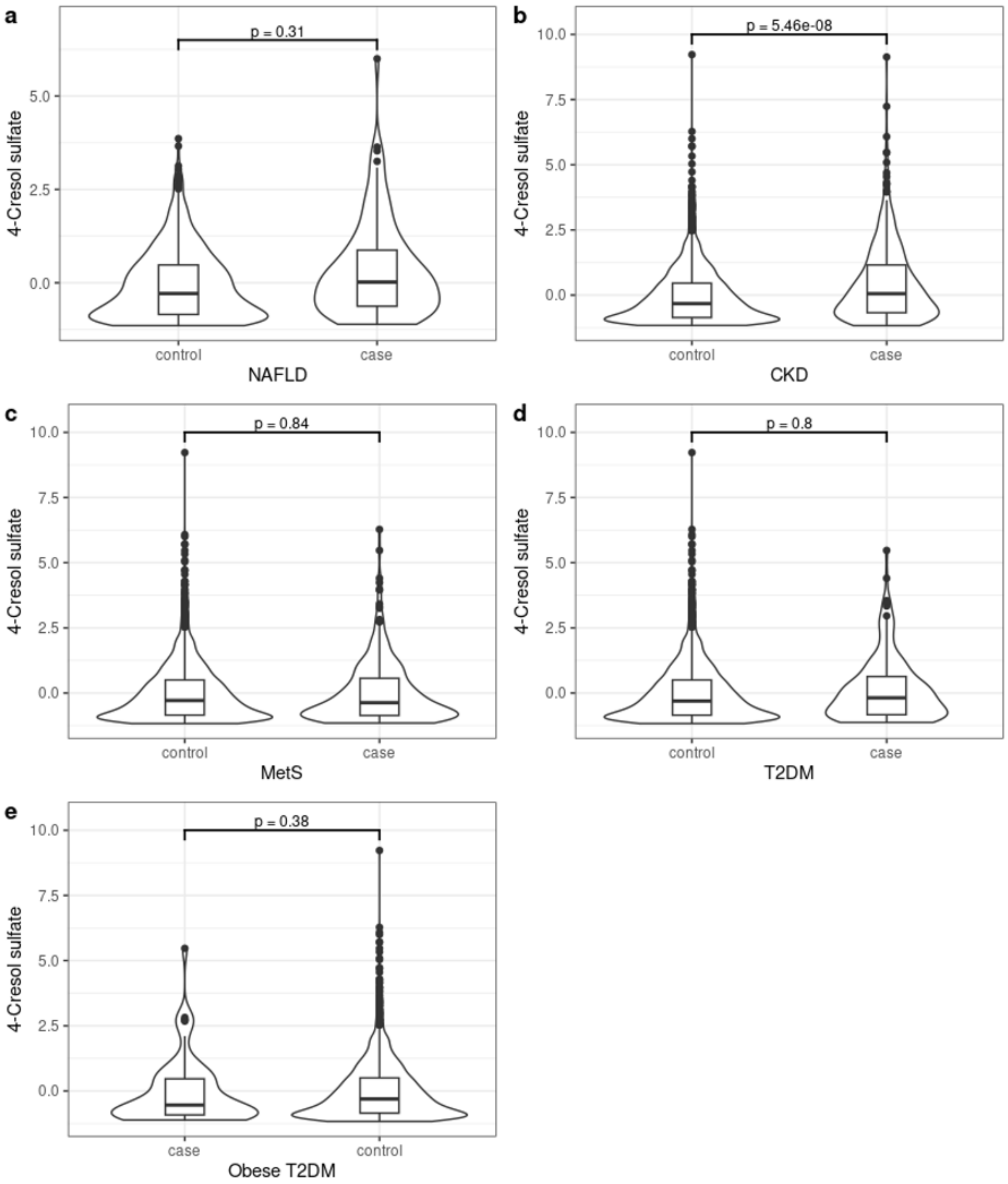

Supplement: Supplementary file 1 — Supplementary Figure S1. [file 41598_2023_40697_MOESM1_ESM.pdf]
