# Supplementary material for: A phenome-wide association study (PheWAS) to identify the health impacts of 4-cresol sulfate in the Nagahama Study
Source: Sci Rep. 2023 Aug 25;13:13926. doi: 10.1038/s41598-023-40697-2 (PMC10457396; doi:10.1038/s41598-023-40697-2)
Supplement: Supplementary file 6 — Supplementary Information [file 41598_2023_40697_MOESM6_ESM.docx]

**Supplementary information**

**Normalization of peak intensities derived from LC-MS**

***Clustering of quality control samples***

Let $q_{i}\left( i=1,2,\ldots, n_{q} \right)$ be a measurement order of $n$ quality controls (QCs) and $a_{q_{i}}$be relative abundance of $q_{i}$. In addition, let $c_{q_{i}}\left( i=1,2,\ldots,n_{q} \right)$ be a class of $q_{i}$ , where measurements clustered into an identical class will be fitted by the same regression model later. The class id is estimated by following algorithms in two steps to mitigate the effects of signal drift and batch bias in the regression process.

QC clustering algorithm

Set window parameters $w_{1}, w_{2}\in N \left( w_{2}<w_{1}<n_{q} \right)$ and thresholds $\theta_{1}, \theta_{2},\theta_{3}\in R^{+}$.­

Step 1

$c_{q_{i}}=0 (i=1,2,\ldots,n_{q})$ , $n_{c}=0$

$$i=1$$

while $i<n_{q}-w_{1}$

Sort $a_{q_{i}},\ldots, a_{q_{i}+w_{1}-1}$ in ascending order and set the result to $a_{1}^{'},\ldots, a_{w_{1}}^{'}$. Let $q_{j}^{'}$be the measurement order of $a_{j}^{'}$.

$$\text{ for }j=w_{2},\ldots,w_{1}-w_{2}$$

$$d_{j}=\min\left( \frac{\left| a_{j+1}-a_{j} \right|}{s_{j}^{1}}, \frac{\left| a_{j+1}-a_{j} \right|}{s_{j}^{2}} \right),$$

$s_{j}^{1}=\sqrt{\frac{1}{j}\sum_{\mathcal{l=}1}^{j} \left( a_{\mathcal{l}}^{'}-\frac{1}{j}\sum_{k=1}^{j} a_{k}^{'} \right)^{2}}$, $s_{j}^{2}=\sqrt{\frac{1}{w_{1}-j}\sum_{\mathcal{l=}j+1}^{w_{1}} \left( a_{\mathcal{l}}^{'}-\frac{1}{w_{1}-j}\sum_{k=j+1}^{w_{1}} a_{k}^{'} \right)^{2}}$

end for $j$

$\hat{d}=\max_{j\in(w_{2},\ldots, w_{1}-w_{2})} d_{j}, t= \underset{j\in(w_{2},\ldots, w_{1}-w_{2})}{\mathrm{argmax}} d_{j}$

$C_{d}=$ $\underset{j\in(0,\ldots,n_{c})}{\mathrm{argmax}} \sum_{\mathcal{l=}1}^{t} \delta(c_{q_{\mathcal{l}}^{'}}, j)$, $C_{u}=\underset{j\in(0,\ldots,n_{c})}{\mathrm{argmax}} \sum_{\mathcal{l=}t+1}^{w_{1}} \delta(c_{q_{\mathcal{l}}^{'}},j)$,

$\delta\left( c,j \right)=\{1 \text{if c=j, 0 if c≠j\} }$

if $C_{d}=0 \cap C_{u}=0$

if $\hat{d}>\theta_{1}\cup\frac{s_{t}^{2}}{s_{t}^{1}}>\theta_{2}$

$$c_{q_{j}^{'}}= n_{c}+1; j\in\left( 1,\ldots,t \right), c_{q_{\mathcal{l}}^{'}}= n_{c}+2; \mathcal{l\in(}t+1,\ldots,w_{1})$$

$$n_{c}= n_{c}+2$$

else

$$c_{q_{j}^{'}}= n_{c}+1, j\in\left( 1,\ldots,w_{1} \right)$$

$n_{c}=$ $n_{c}+1$

end if

else if $C_{d}=0$

if $\hat{d}>\theta_{1}\cup\frac{s_{t}^{2}}{s_{t}^{1}}>\theta_{2}$

$$n_{c}=n_{c}+1$$

$$c_{q_{j}^{'}}=n_{c}; j\in\left( 1,\ldots,t \right), c_{q_{\mathcal{l}}^{'}}=C_{u}\mathcal{; l\in(}t+1,\ldots,w_{1})$$

else

$$c_{q_{j}^{'}}=C_{u}, j\in\left( 1,\ldots,w_{1} \right)$$

end if

else if $C_{u}=0$

if $\hat{d}>\theta_{1}$

$$n_{c}=n_{c}+1$$

$$c_{q_{j}^{'}}=C_{d}; j\in\left( 1,\ldots,t \right), c_{q_{\mathcal{l}}^{'}}=n_{c}\mathcal{; l\in(}t+1,\ldots,w_{1})$$

else

$$c_{q_{j}^{'}}=C_{d}, j\in\left( 1,\ldots,w_{1} \right)$$

end if

else

if $\hat{d}>\theta_{1}\cup\frac{s_{t}^{2}}{s_{t}^{1}}>\theta_{2}$

if $C_{d}\neq C_{u}$

$$c_{q_{j}^{'}}=C_{d}; j\in\left( 1,\ldots,t \right), c_{q_{\mathcal{l}}^{'}}=C_{u}\mathcal{; l\in(}t+1,\ldots,w_{1})$$

else

$n_{c}=n_{c}+1$

$C=$ $\underset{j\in(1,\ldots,n_{c})}{\mathrm{argmax}} \sum_{\mathcal{l=}i-w_{2}}^{i-1} \delta(c_{q_{\mathcal{l}}}, j)$

$$m_{d}= \frac{1}{{tw}_{2}} \sum_{\mathcal{l=}i-w_{2}}^{i-1} \sum_{j=1}^{t} \left| a_{q_{\mathcal{l}}}-a_{q_{j}^{'}} \right|$$

$$m_{u}= \frac{1}{{(w_{1}-t)w}_{2}} \sum_{\mathcal{l=}i-w_{2}}^{i-1} \sum_{j=t+1}^{w_{1}} \left| a_{q_{\mathcal{l}}}-a_{q_{j}^{'}} \right|$$

if $m_{d}<m_{u}$

$$c_{q_{j}^{'}}=C; j\in\left( 1,\ldots,t \right), c_{q_{\mathcal{l}}^{'}}=n_{c}\mathcal{; l\in(}t+1,\ldots,w_{1})$$

else

$$c_{q_{j}^{'}}=n_{c}; j\in\left( 1,\ldots,t \right), c_{q_{\mathcal{l}}^{'}}=C\mathcal{; l\in(}t+1,\ldots,w_{1})$$

end if

end if

else

$$c_{q_{j}^{'}}=C_{d}; j\in\left( 1,\ldots,t \right), c_{q_{\mathcal{l}}^{'}}=C_{u}\mathcal{; l\in(}t+1,\ldots,w_{1})$$

end if

$i=i+w_{2}$

loop

Step 2

$$k_{i}=\sum_{j=1}^{n_{q}} \delta(c_{q_{j}}, i);i=1,\ldots,n_{c}$$

$$\left\{ b_{j}^{i} \right| {j=1,\ldots,k_{i}, b}_{x}^{i}<b_{y}^{i} \text{for} x<y\}=\left\{ \mathcal{l\in(}1,\ldots,n_{q})| \delta\left( C_{q_{\mathcal{l}}},i \right)=1 \right\}$$

$$n_{c}^{'}=n_{c}$$

for $i=1,\ldots,n_{c}'$

if $k_{i}\leq{2w}_{2}$

continue

end if

$m_{c}=\frac{1}{k_{i}-1}\sum_{j=2}^{k_{i}} \left| a_{q_{b_{j}^{i}}}-a_{q_{b_{j-1}^{i}}} \right|, s_{c}=\sqrt{\frac{1}{k_{i}-1}\sum_{j=2}^{k_{i}} \left( a_{q_{b_{j}^{i}}}-a_{q_{b_{j-1}^{i}}}-m_{c} \right)^{2}}$

for $j=w_{2},\ldots, k_{i}-w_{2}$

if $\max\left( \max_{x\in(j-w_{2}+2,\ldots, j)}\left( b_{x}^{i}-b_{x-1}^{i} \right), \max_{x\in(j+2,{\ldots,j+w}_{2})}\left( b_{x}^{i}-b_{x-1}^{i} \right) \right)>w_{2}$

$v_{j}=0$

continue

end if

$d_{1}=\frac{1}{w_{2}-1}\sum_{x=j-w_{2}+2}^{j} \left| a_{q_{b_{x}^{i}}}-a_{q_{b_{x-1}^{i}}} \right|, d_{2}=\frac{1}{w_{2}-1}\sum_{x=j+2}^{j+w_{2}} \left| a_{q_{b_{x}^{i}}}-a_{q_{b_{x-1}^{i}}} \right|$

if $d_{1}\geq d_{2}>0$

$v_{j}=\frac{\left| a_{q_{b_{j}^{i}}}-a_{q_{b_{j+1}^{i}}} \right|-d_{2}}{S_{c}}$

else if $d_{2}>d_{1}>0$

$v_{j}=\frac{\left| a_{q_{b_{j}^{i}}}-a_{q_{b_{j+1}^{i}}} \right|-d_{1}}{S_{c}}$

end if

end for $j$

for $j=w_{2},\ldots, k_{i}-w_{2}$

if $v_{j}>\theta_{3}$

if $\max_{\mathcal{l\in(}j-w_{2},\ldots,j+w_{2})} v_{\mathcal{l}}=v_{j}$

$n_{c}=n_{c}+1$

$$c_{q_{\mathcal{l}}}=n_{c}\mathcal{;l\in}\left( j+1,\ldots,k_{i} \right)$$

end if

end if

end for $j$

end for $i$

We conducted the above clustering steps with parameters $w_{1}=20, w_{2}=4, \theta_{1}= \theta_{2}= \theta_{3}= 3.0$ in this study.

***Regression of quality control data***

Quality control data clustered to $n_{c}$ classes are fitted independently for each class. Let $Q_{c}=\{q_{i}|c_{q_{i}}=c, i=1,\ldots,k_{c},q_{\mathcal{l}}<q_{m} \text{for }\mathcal{l<}m\}$ be set of QCs belonging to class $c$ and let $A_{c}=\{a_{q_{i}}\}$ be relative abundances of $Q_{c}$. Relative abundances $A_{c}$ on $Q_{c}$ are regressed by using support vector regression. For the regression, we set linear and Gaussian kernels defined by:

Linear kernel: $K\left( q_{j},q_{\mathcal{l}} \right)=q_{j}q_{\mathcal{l}}$

Gaussian kernel: $K\left( q_{j},q_{\mathcal{l}} \right)=exp\left( -\frac{\left( q_{j}-q_{\mathcal{l}} \right)^{2}}{2\sigma^{2}} \right)$.

The kernel functions were estimated with soft margin $\xi$ by using a grid search (for $\sigma$ and $\xi$, 20 points were evenly spaced on a logarithmic scale in the intervals of $\left[ {10}^{-4}, 1 \right]$ and $\left[ {10}^{-2},10 \right],$ respectively) and 5-fold cross validation. Let $f_{c}$ be the optimal linear or Gaussian kernel function for class $c$.

***Intensity normalization of cohort samples***

Let $s_{i}$ and $v_{i}(i=1,\ldots, n_{m})$be a measurement order and its relative abundance of $n_{m}$ cohort samples, respectively. Let $S_{c}=\{s_{i}|\min\left\{ q\in Q_{c} \right\}<s_{i}<\max\{q\in Q_{c}\}\}$ be cohort samples within class $c$. Next, calculate mean and standard deviation of $a_{q}$ in cluster $c$ by

$m_{Q_{c}}= \frac{1}{n_{Q_{c}}}\sum_{q_{i}\in Q_{c}} a_{q_{i}}, \sigma_{Q_{c}}=\sqrt{\frac{1}{n_{Q_{c}}}\sum_{q_{i}\in Q_{c}} \left( a_{q_{i}}-m_{Q_{c}} \right)^{2}}$ ,

where $n_{Q_{c}}$ is the number of samples in $Q_{c}$. Then, weighted target variable $t_{i}$ is calculated by

$t_{i}=\left\{ \begin{aligned} &f_{c}\left( s_{i} \right) if ⋕\left\{ c | S_{c}\ni s_{i} \right\}=1, \\ & \sum_{c|S_{c}\ni s_{i}} f_{c}\left( s_{i} \right)\frac{w_{i}-\frac{v_{s_{i}}-f_{c}(s_{i})}{\sigma_{Q_{c}}}}{w_{i}} else, \end{aligned} \right.$

, where $w_{i}=\sum_{c|S_{c}\ni s_{i}} \frac{v_{s_{i}}-f_{c}\left( s_{i} \right)}{\sigma_{Q_{c}}}.$ Finally, relative abundance $v_{i}$ is normalized to $y_{i}$ by the following equation:

$y_{i}= \frac{\alpha v_{i}+\beta v_{i}^{2}+\gamma}{t_{i}+\gamma}m_{a}, m_{a}= \frac{1}{n_{m}}\sum_{i=1}^{n_{m}} v_{i}$.

Parameters $\alpha, \beta, \gamma$ are determined in the following steps. First, samples were divided into five sets of $S_{V}^{1}\{s_{1},\ldots,s_{\left\lfloor\frac{n_{m}}{5} \right\rfloor}\}$, $S_{V}^{2}\{s_{\left\lfloor\frac{n_{m}}{5} \right\rfloor+1},\ldots,s_{\left\lfloor\frac{2n_{m}}{5} \right\rfloor}\}$, $S_{V}^{3}\{s_{\left\lfloor\frac{{2n}_{m}}{5} \right\rfloor+1},\ldots,s_{\left\lfloor\frac{3n_{m}}{5} \right\rfloor}\}$, $S_{V}^{4}\{s_{\left\lfloor\frac{{3n}_{m}}{5} \right\rfloor+1},\ldots,s_{\left\lfloor\frac{4n_{m}}{5} \right\rfloor}\}$, $S_{V}^{5}\{s_{\left\lfloor\frac{{4n}_{m}}{5} \right\rfloor+1},\ldots,s_{n_{m}}\}$. Let $y_{k}^{25}(\alpha, \beta, \gamma) \mathrm{and} y_{k}^{75}(\alpha, \beta, \gamma)$ be 0.25 and 0.75 quantiles of $y$ values in $S_{V}^{k}$ for $\alpha, \beta\mathrm{and} \gamma$, respectively. Then parameters are determined to optimize the following equation:

$\hat{\alpha}, \hat{\beta}, \hat{\gamma}=\underset{\alpha, \beta,\gamma}{\mathrm{argmax}} \sum_{k=1}^{4} \sum_{\mathcal{l=}k+1}^{5} \left\{ \max\left( u_{k\mathcal{,l}}(\alpha,\beta,\gamma), 0 \right)+\max\left( u_{\mathcal{l,}k}(\alpha,\beta,\gamma), 0 \right) \right\}$,

$$u_{k\mathcal{,l}}\left( \alpha,\beta,\gamma\right)=\frac{\min\left( y_{k}^{75}\left( \alpha,\beta,\gamma\right),y_{\mathcal{l}}^{75}(\alpha,\beta,\gamma) \right)-\max\left( y_{k}^{25}\left( \alpha,\beta,\gamma\right),y_{\mathcal{l}}^{25}(\alpha,\beta,\gamma) \right)}{y_{k}^{75}(\alpha, \beta,\gamma)-y_{k}^{25}(\alpha,\beta,\gamma)}.$$

The grid search with intervals of $\alpha\in\left[ 0, 2.0 \right] with a step of 0.01, \beta\in[0, 1.0]$ with a step of 0.02, and $\gamma\in[0,m_{a}]$ with a step of 0.02$m_{a}$ was conducted to determine the parameters.
